# Dose-escalation of human anti-interferon-α receptor monoclonal antibody MEDI-546 in subjects with systemic sclerosis: a phase 1, multicenter, open label study

**DOI:** 10.1186/ar4492

**Published:** 2014-02-24

**Authors:** Avram Goldberg, Thomas Geppert, Elena Schiopu, Tracy Frech, Vivien Hsu, Robert W Simms, Stanford L Peng, Yihong Yao, Nairouz Elgeioushi, Linda Chang, Bing Wang, Stephen Yoo

**Affiliations:** 1 North Shore LIJ Health Systems and Division of Rheumatology, Hofstra North Shore-LIJ School of Medicine, Lake Success, NY, USA; 2 Metroplex Clinical Research Center, LLC, Dallas, TX, USA; 3 University of Michigan, Ann Arbor, MI, USA; 4 University of Utah, Salt Lake City, UT, USA; 5 RWJ Medical School Clinical Research Center, New Brunswick, NJ, USA; 6 Boston University School Of Medicine, Boston, MA, USA; 7 Benaroya Research Institute at Virginia Mason Medical Center, Seattle, WA, USA; 8 MedImmune, Gaithersburg, MD, USA; 9 MedImmune, Hayward, CA, USA

## Abstract

**Introduction:**

Type I interferons (IFNs) are implicated in the pathogenesis of systemic sclerosis (SSc). MEDI-546 is an investigational human monoclonal antibody directed against the type I IFN receptor. This Phase 1 study evaluated the safety/tolerability, pharmacokinetics (PK), immunogenicity, and pharmacodynamics (PD) of single and multiple intravenous doses of MEDI-546 in adults with SSc.

**Methods:**

Subjects (≥18 years) with SSc were enrolled in an open-label, dose-escalation study to receive single (0.1, 0.3, 1.0, 3.0, 10.0, or 20.0 mg/kg), or 4 weekly intravenous doses (0.3, 1.0, or 5.0 mg/kg/week) of MEDI-546. Subjects were followed for 12 weeks. Safety assessments included adverse events (AEs), laboratory results, and viral monitoring. Blood samples were collected from all subjects for determination of PK, presence of anti-drug antibodies (ADAs), and expression of type I IFN-inducible genes.

**Results:**

Of 34 subjects (mean age 47.4 years), 32 completed treatment and 33 completed the study. Overall, 148 treatment-emergent AEs (TEAEs) were reported (68.9% mild, 27.7% moderate). TEAEs included one grade 1 infusion reaction (5.0 mg/kg/week multiple dose). Of 4 treatment-emergent serious AEs (skin ulcer, osteomyelitis, vertigo, and chronic myelogenous leukemia (CML)), only CML (1.0 mg/kg/week multiple dose) was considered possibly treatment-related. MEDI-546 exhibited non-linear PK at lower doses. ADAs were detected in 5 subjects; no apparent impact on PK was observed. Peak inhibition of the type I IFN signature in whole blood was achieved within 1 day and in skin after 7 days.

**Conclusion:**

The safety/tolerability, PK, and PD profiles observed in this study support further clinical development of MEDI-546.

**Trial Registration:**

ClinicalTrials.gov NCT00930683

## Introduction

Systemic sclerosis (SSc) is an autoimmune multisystem disease of unknown etiology, characterized by structural abnormalities in small blood vessels and excessive deposition of extracellular matrix components [1,2]. Patients with diffuse SSc have a greater likelihood of organ damage, reduced quality of life, and long-term morbidity and mortality, leading to a high economic and patient burden [3,4].

Current therapies for SSc are generally aimed at controlling symptoms, and do not address the underlying causes of the disease [5]. A recent report from the German Network for Systemic Scleroderma showed that 41% of patients with SSc were treated with corticosteroids and 36% received immunosuppressive agents, despite a lack of robust evidence demonstrating the efficacy of these treatments [6]. High-dose corticosteroid therapy (≥15 mg/day) has been associated with the development of renal crisis, a life-threatening disease manifestation of SSc [7]. Although immunosuppressive therapy has demonstrated some efficacy in clinical studies, it does not appear to provide benefits during later phases of SSc, and long-term usage is limited by its potential toxicity [5]. Currently, there are no effective disease-modifying treatments available for patients with SSc [8]. Considering the high mortality of SSc, there is a significant unmet need for novel therapies that clearly control or alter the aberrant fibrotic pathways of the disease, with acceptable toxicities [9].

An increasing body of evidence suggests that type I interferons (IFNs), may play a role in SSc pathogenesis [10]. In some studies, elevated levels of type I IFNs have been observed in the blood of patients with SSc [11,12]. In addition, increased expression of type I IFN-induced genes and proteins has been observed in the blood and skin of patients with SSc [13-17]. Furthermore, IFN therapy has been implicated in the development or exacerbation of SSc or sclerodermatous-like disease [18,19]. These studies indicate that the type I IFN pathway is activated in patients with SSc and that these patients may benefit from anti-IFN therapy.

All type I IFNs bind to the same heterodimeric type I IFNα receptor (IFNAR), comprising subunits IFNAR1 and IFNAR2 [20,21]. MEDI-546 is an investigational human immunoglobulin G1 kappa monoclonal antibody directed against IFNAR1. By blocking type I IFN-mediated signaling, MEDI-546 suppresses the receptor-mediated biological activity of all type I IFNs (unpublished results).

In this study, the safety profile (primary objective) and pharmacokinetics (PK), immunogenicity, and pharmacodynamics (PD) (secondary objectives) of single and multiple intravenous (IV) doses of MEDI-546 were examined in subjects with SSc.

## Methods

### Study design

This was a Phase 1, multicenter, open-label, dose-escalation study of single and multiple IV doses of MEDI-546 in adult subjects with SSc. The study is registered on ClinicalTrials.gov (Study MI-CP180; NCT00930683). The study protocol, protocol amendments, and subject informed consent documents were approved by Institutional Review Boards (IRBs). A list of the IRBs is provided below. Written informed consent was obtained from all subjects before study entry or any study-specific activities were carried out. An electronic data capture system was utilized for this study.

The primary objective was to evaluate the safety and tolerability of single and multiple IV escalating doses of MEDI-546. Secondary objectives of the study were to assess the PK, immunogenicity potential, and PD of MEDI-546.

**Figure 1:**
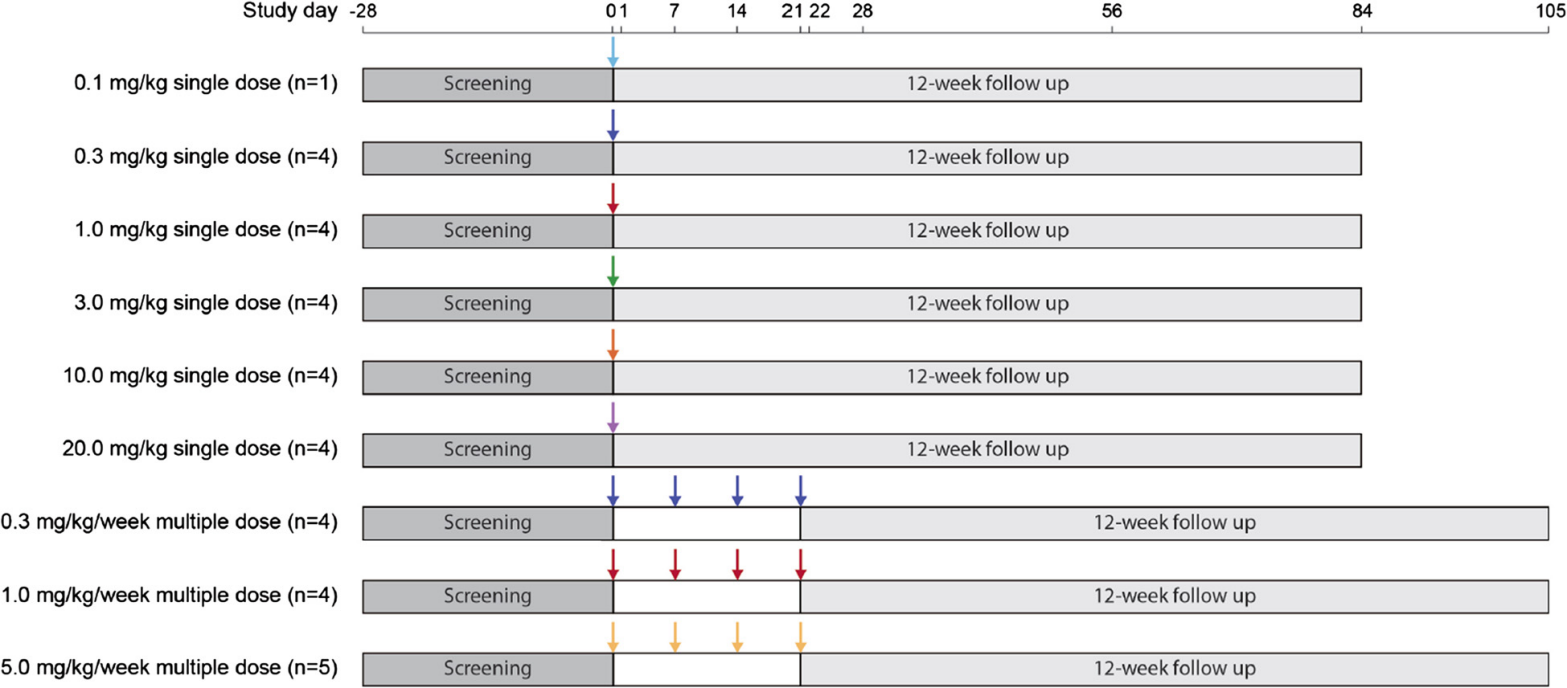
Study design.

Following a 28-day screening period, eligible subjects were randomized into 9 groups: 6 groups received 1 of 6 single MEDI-546 doses (0.1, 0.3, 1.0, 3.0, 10.0, or 20.0 mg/kg) sequentially, and 3 groups received 1 of 3 multiple MEDI-546 doses (0.3, 1.0, or 5.0 mg/kg/week, 4 doses in total) (Figure 1). The single doses were started at 0.1 mg/kg. The single doses were escalated sequentially. For single-dose groups ≤10.0 mg/kg, dose escalation decisions were made based on the review of safety data up to day 7 in the preceding lower dose group. The 20.0 mg/kg single-dose group and all multiple-dose groups were enrolled simultaneously, after the cumulative safety data for the lower single doses were approved. MEDI-546 was administered as IV infusion over ≥60 minutes. Administration occurred on day 0 for single-dose groups, with follow-up until day 84, and on days 0, 7, 14, and 21 for multiple-dose groups, with follow-up until day 105. Evaluations were scheduled on study days 0, 1, 7, 14, 21, 28, 56, and 84 for single-dose groups and, additionally, on days 22 and 105 for multiple-dose groups.

### Subjects

Adults aged ≥18 years who met the American College of Rheumatology preliminary criteria for SSc [22] were enrolled in the study. All study participants were required to have at least moderate skin thickening (≥2 by modified Rodnan Skin Score [mRSS]) in an area suitable for repeat biopsy. Stable concomitant medication to treat SSc or other medical conditions was allowed, unless the treatment met the following exclusion criteria. Subjects were excluded if they had received any of the following medications within 28 days before study day 0: cyclophosphamide, systemic cyclosporine, or thalidomide. Subjects were excluded if they had received fluctuating doses or in excess of permitted doses of antimalarials (hydroxychlorquine >600 mg/day), mycophenolate mofetil (>3 g/day), methotrexate (>25 mg/week), leflunomide (>20 mg/day within 6 months of study entry), or azathioprine (>3 mg/kg/week); any antiviral treatment; or an investigational treatment within 28 days of study day 0. Also excluded were subjects who had received prednisone (>20 mg/day or fluctuating doses) or nonsteroidal anti-inflammatory drugs (fluctuating doses) within 14 days of study day 0. Other exclusions included history of primary immunodeficiency, severe viral infection, herpes zoster infection, cancer, and alcohol or drug abuse.

### Safety and tolerability

Adverse events (AEs) and serious AEs (SAEs) were monitored at screening and at each scheduled evaluation visit. AEs and SAEs were graded on a scale of 1–5 using the National Cancer Institute Common Terminology Criteria for Adverse Events, version 4.03 [23] and by relationship to study drug. Grade refers to the severity of the AE: grade 1: mild AE; grade 2: moderate AE; grade 3: severe AE; grade 4: life-threatening or disabling AE; grade 5: death related to AE.

Hematology, serum chemistry, and urinalysis were assessed at screening and at each scheduled evaluation visit, except for study days 1 and 22 (multiple doses only). Viral monitoring was performed on days 0 (before dosing), 28, 56, 84, and 105. Surveillance was performed for cytomegalovirus (CMV), Epstein-Barr virus (EBV), herpes simplex virus (HSV-1 and HSV-2), and human papilloma virus (HPV). Oropharyngeal and vaginal swabs were cultured for HSV-1 and HSV-2, vaginal swabs were tested for HPV, and blood samples were tested for CMV and EBV. HPV DNA testing was performed using the Hybrid Capture 2 High-Risk assay (HC2), with a sensitivity and specificity range of 81–93% and 80–94%, respectively [24]. CMV, EBV and HSV testing were performed at ACM Global Central Laboratory (Rochester, NY, USA). CMV and EBV were detected using quantitative real-time polymerase chain reaction assays. HSV was identified by its characteristic cytopathic effect; virus typing was accomplished using fluorescent antibodies against HSV-1 and HSV-2.

### Pharmacokinetics

Blood samples for PK assessments were collected at each scheduled evaluation visit. Serum concentrations of MEDI-546 were measured using a validated electrochemiluminescent (ECL) assay. In this assay, biotinylated IFNAR1 and a sulfo-TAG labeled monoclonal antibody specific for MEDI-546 were utilized as the capturing and detecting reagents, respectively. The quantitation range of the assay was 20–1280 ng/mL, for 1:10 diluted human serum.

### Immunogenicity

Blood samples for anti-drug antibody (ADA) assessments were taken on days 0 (pre-dose), 28, 56, 84, and 105. The presence of ADAs in human serum samples was determined using an ECL, solution-phase, bridging immunoassay. Positive titers, expressed as reciprocal dilutions, were defined as 1:30 or higher.

Auto-antibody levels, including antinuclear antibody and anti-topoisomerase I (anti-SCL-70), were measured on day 0, as part of the baseline SSc evaluation.

### Pharmacodynamics

Five type I IFN-inducible genes (RSAD2, IFI44, IFI44L, IFI27, IFI6) were selected as pharmacodynamics markers for MEDI-546 based on their prevalence and magnitude of overexpression in patients with SSc as compared with healthy controls [25,26]. Total RNA was extracted from blood and skin biopsies using the PAXgene Blood RNA kit and the Qiagen RNeasy Fibrous Tissue Mini kit (Hilden, Germany), respectively. RNA purity and concentration were determined spectrophotometrically (260/280 > 1.9). RNA quality was assessed on an Agilent 2100 Bioanalyzer using the RNA 6000 Nano LabChip® (Santa Clara, CA, USA).

The expression level of the 5-gene marker in whole blood and skin was measured using Affymetrix Human Genome U133 Plus arrays (Santa Clara, CA, USA) [25]. Blood samples were procured at each evaluation visit, except for days 21 and 22, and skin biopsies were collected on days 0, 7 (single doses only), and 28 (multiple doses only). Transcript profiling was conducted according to standard methods [27]. The rationale for the definition of positive gene signatures as >2.9 for skin and >1.8 for blood and the details of score generation are included in the Wang publication [26].

### Statistical methods

No formal sample size calculation was performed, as the primary endpoint was safety and tolerability. A sample size of 33 subjects was planned, with 53 being the maximum number of subjects allowed in the study. The safety population, comprising all subjects who received MEDI-546, was used for safety and tolerability evaluations and for measurements of PD; the evaluable population for PK included all subjects in the safety population with ≥1 valid MEDI-546 serum concentration assessment; the evaluable population for immunogenicity included all subjects in the safety population with ≥1 valid immunogenicity test result post dose.

No formal statistical hypothesis testing was planned for the primary endpoint. Noncompartmental PK data analysis was performed using WinNonlin Professional version 5.2 (Pharsight Corp, St Louis, MO, USA). The maximum serum concentration (C_max_), area under the serum concentration-time curve from 0 to infinity (AUC_inf_; single doses only), AUC from time 0 to 7 days post dose administration (AUC_0–7_; multiple doses only), serum elimination half-life (t_1/2_), and systemic clearance (CL) were calculated and reported. Incidences of positive ADA were summarized for each dose group and all groups combined.

Type I IFN gene signature scores were calculated as median-fold change in the expression of 5 IFN-inducible genes in SSc subjects, with respect to a pooled panel of healthy volunteers. Positive gene signature scores were defined as >2.9 in whole blood and >1.8 in skin, using blood and skin specimens from 54 and 30 healthy volunteers, respectively (data on file). Subjects were divided into signature positive and signature negative groups based on type I IFN signature scores at baseline.

## Results

### Baseline characteristics

The study started on 19 August 2009 and was completed on 14 July 2011. Thirty-four subjects from 7 US sites were enrolled, of which 33 subjects completed the study, with 1 subject in the 5.0 mg/kg/week multiple-dose group discontinuing from the study for other reasons. Most subjects were female (79.4%), white (73.5%), and had diffuse cutaneous SSc (94.1%) and Raynaud’s phenomenon (97.1%). Mean mRSS was 23.3. Overall, 22 subjects (64.7%) had positive type I IFN gene signature in whole blood, 15 (44.1%) in skin, and 7 (20.6%) had a history of viral reactivation. Positive antinuclear antibodies and anti-SCL-70 antibodies were found in 24 (70.6%) and 11 (32.4%) subjects, respectively. The most common medications used at baseline were corticosteroids ≤10 mg/day (n = 12; 35.3%) and methotrexate (n = 11; 32.4%) for >6 months. Baseline demographic and disease characteristics by MEDI-546 dose group are summarized in Table 1.

**Table 1:** Baseline demographics and disease characteristics

**Characteristic**	**MEDI-546**									
	**0.1 mg/kg single dose**	**0.3 mg/kg single dose**	**1.0 mg/kg single dose**	**3.0 mg/kg single dose**	**10.0 mg/kg single dose**	**20.0 mg/kg single dose**	**0.3 mg/kg/week multiple dose**	**1.0 mg/kg/week multiple dose**	**5.0 mg/kg/week multiple dose**	**Total**
	**(n = 1)**	**(n = 4)**	**(n = 4)**	**(n = 4)**	**(n = 4)**	**(n = 4)**	**(n = 4)**	**(n = 4)**	**(n = 5)**	**(N = 34)**
Age, mean (SD), years	41.0 (NA)	58.3 (10.0)	45.0 (7.1)	33.0 (10.4)	48.0 (12.5)	45.5 (9.5)	42.5 (11.4)	49.3 (9.5)	57.2 (12.3)	47.4 (12.0)
Female, n (%)	1 (100.0)	3 (75.0)	4 (100.0)	3 (75.0)	4 (100.0)	3 (75.0)	2 (50.0)	3 (75.0)	4 (80.0)	27 (79.4)
White, n (%)	1 (100.0)	3 (75.0)	2 (50.0)	2 (50.0)	2 (50.0)	3 (75.0)	4 (100.0)	4 (100.0)	4 (80.0)	25 (73.5)
Weight, mean (SD), kg	63.5 (NA)	57.7 (5.4)	71.7 (26.8)	72.8 (30.1)	73.4 (28.2)	68.7 (13.6)	87.8 (11.6)	75.9 (33.7)	72.2 (21.8)	72.3 (21.8)
Diffuse cutaneous systemic SSc, n (%)	1 (100.0)	4 (100.0)	4 (100.0)	4 (100.0)	4 (100.0)	4 (100.0)	3 (75.0)	4 (100.0)	4 (80.0)	32 (94.1)
Raynaud’s, n (%)	1 (100.0)	4 (100.0)	4 (100.0)	4 (100.0)	4 (100.0)	4 (100.0)	3 (75.0)	4 (100.0)	5 (100.0)	33 (97.1)
mRSS, mean (SD)	16.0 (NA)	24.5 (6.6)	23.0 (11.0)	21.0 (2.4)	22.0 (10.4)	31.8 (12.2)	17.5 (5.1)	24.5 (10.1)	23.8 (7.9)	23.3 (8.6)
Positive type I IFN gene signature^a^										
Whole blood, n (%)	0 (0.0)	4 (100.0)	2 (50.0)	3 (75.0)	3 (75.0)	3 (75.0)	1 (25.0)	3 (75.0)	3 (60.0)	22 (64.7)
Skin, n (%)	0 (0.0)	2 (50.0)	3 (75.0)	1 (25.0)	4 (100.0)	1 (25.0)	0 (0.0)	3 (75.0)	1 (20.0)	15 (44.1)
History of viral reactivation, n (%)	0 (0.0)	1 (25.0)	0 (0.0)	0 (0.0)	0 (0.0)	2 (50.0)	1 (25.0)	2 (50.0)	1 (20.0)	7 (20.6)
Positive antinuclear antibody, n (%)	1 (100.0)	2 (50.0)	4 (100.0)	4 (100.0)	3 (75.0)	2 (50.0)	3 (75.0)	3 (75.0)	2 (40.0)	24 (70.6)
Positive anti-SCL-70 antibody, n (%)	1 (100.0)	2 (50.0)	0 (0.0)	3 (75.0)	1 (25.0)	1 (25.0)	2 (50.0)	0 (0.0)	1 (20.0)	11 (32.4)
Corticosteroids ≤10 mg/day										
for <6 months, n (%)	0 (0.0)	1 (25.0)	2 (50.0)	0 (0.0)	1 (25.0)	0 (0.0)	0 (0.0)	0 (0.0)	0 (0.0)	4 (11.8)
for >6 months, n (%)	0 (0.0)	0 (0.0)	2 (50.0)	3 (75.0)	1 (25.0)	3 (75.0)	0 (0.0)	1 (25.0)	2 (40.0)	12 (35.3)
Corticosteroids >10 and ≤20 mg/day										
for <6 months, n (%)	0 (0.0)	1 (25.0)	1 (25.0)	0 (0.0)	0 (0.0)	0 (0.0)	0 (0.0)	0 (0.0)	0 (0.0)	2 (5.9)
for >6 months, n (%)	0 (0.0)	1 (25.0)	0 (0.0)	2 (50.0)	2 (50.0)	1 (25.0)	0 (0.0)	0 (0.0)	0 (0.0)	6 (17.6)
Antimalarials for >6 months, n (%)	0 (0.0)	0 (0.0)	0 (0.0)	1 (25.0)	1 (25.0)	2 (50.0)	0 (0.0)	1 (25.0)	0 (0.0)	5 (14.7)
Methotrexate										
for <6 months, n (%)	0 (0.0)	0 (0.0)	1 (25.0)	0 (0.0)	0 (0.0)	0 (0.0)	1 (25.0)	0 (0.0)	0 (0.0)	2 (5.9)
for >6 months, n (%)	0 (0.0)	1 (25.0)	1 (25.0)	3 (75.0)	2 (50.0)	2 (50.0)	0 (0.0)	1 (25.0)	1 (20.0)	11 (32.4)

^a^Positive type I IFN gene signature was defined as a baseline (day 0 pre-dose) fold change value >2.9 in whole blood and >1.8 in skin.

*IFN* interferon, *mRSS* modified Rodnan Skin Score, *SD* standard deviation, *SSc* systemic sclerosis.

All 34 subjects were included in the safety population and the evaluable population for PK. The population for ADA comprised 32 subjects; 2 subjects in the 5.0 mg/kg/week multiple-dose group were excluded for discontinuing treatment.

### Safety and tolerability assessments

In total, 148 treatment-emergent AEs (TEAEs) were reported during the study, with all 34 subjects reporting ≥1 TEAE. The most frequently reported TEAEs (incidence >10% of total population) were upper respiratory tract infection (n = 10; 29.4%), headache (n = 7; 20.6%), diarrhea (n = 6; 17.6%), nausea (n = 6; 17.6%), arthralgia (n = 4; 11.8%), fatigue (n = 4; 11.8%), and pruritus (n = 4; 11.8%) (Table 2). Most TEAEs were grade 1 (68.9%) or grade 2 (27.7%) in intensity. Two grade 3 TEAEs were reported in the 3.0 mg/kg single-dose group (osteomyelitis and skin ulcer) and 3 in the 5.0 mg/kg/week multiple-dose group (vertigo, musculoskeletal chest pain, and musculoskeletal pain), and were considered unrelated to treatment. No relationship was apparent between MEDI-546 dose and nature, frequency, or severity of TEAEs reported.

**Table 2:** **Frequently reported (>10**% **overall and >1 subject in any group) treatment-emergent adverse events and laboratory abnormalities**

	**MEDI-546**									
	**0.1 mg/kg single dose**	**0.3 mg/kg single dose**	**1.0 mg/kg single dose**	**3.0 mg/kg single dose**	**10.0 mg/kg single dose**	**20.0 mg/kg single dose**	**0.3 mg/kg/week multiple dose**	**1.0 mg/kg/week multiple dose**	**5.0 mg/kg/week multiple dose**	**Total**
	**(n = 1)**	**(n = 4)**	**(n = 4)**	**(n = 4)**	**(n = 4)**	**(n = 4)**	**(n = 4)**	**(n = 4)**	**(n = 5)**	**(N = 34)**
**TEAEs**										
Total number of TEAEs	1	10	15	15	18	11	20	20	38	148
Total subjects reporting ≥1 TEAEs, n (%)	1 (100.0)	4 (100.0)	4 (100.0)	4 (100.0)	4 (100.0)	4 (100.0)	4 (100.0)	4 (100.0)	5 (100.0)	34 (100.0)
Upper respiratory tract infection, n (%)^a^	0 (0.0)	2 (50.0)	1 (25.0)	2 (50.0)	0 (0.0)	2 (50.0)	0 (0.0)	3 (75.0)	0 (0.0)	10 (29.4)
Headache, n (%)^a^	0 (0.0)	1 (25.0)	0 (0.0)	0 (0.0)	2 (50.0)	1 (25.0)	2 (50.0)	1 (25.0)	0 (0.0)	7 (20.6)
Diarrhea, n (%)^a^	0 (0.0)	0 (0.0)	3 (75.0)	0 (0.0)	0 (0.0)	1 (25.0)	0 (0.0)	1 (25.0)	1 (20.0)	6 (17.6)
Nausea, n (%)^a^	0 (0.0)	0 (0.0)	0 (0.0)	1 (25.0)	2 (50.0)	1 (25.0)	0 (0.0)	1 (25.0)	1 (20.0)	6 (17.6)
Arthralgia, n (%)^a^	0 (0.0)	0 (0.0)	1 (25.0)	0 (0.0)	1 (25.0)	0 (0.0)	1 (25.0)	1 (25.0)	0 (0.0)	4 (11.8)
Fatigue, n (%)^a^	0 (0.0)	0 (0.0)	1 (25.0)	0 (0.0)	1 (25.0)	0 (0.0)	1 (25.0)	1 (25.0)	0 (0.0)	4 (11.8)
Pruritus, n (%)^a^	0 (0.0)	0 (0.0)	1 (25.0)	0 (0.0)	1 (25.0)	0 (0.0)	0 (0.0)	1 (25.0)	1 (20.0)	4 (11.8)
Dizziness, n (%)^a^	0 (0.0)	0 (0.0)	1 (25.0)	0 (0.0)	0 (0.0)	0 (0.0)	0 (0.0)	0 (0.0)	2 (40.0)	3 ( 8.8)
Musculoskeletal chest pain, n (%)^a^	0 (0.0)	0 (0.0)	1 (25.0)	0 (0.0)	0 (0.0)	0 (0.0)	0 (0.0)	0 (0.0)	2 (40.0)	3 ( 8.8)
Urticaria, n (%)^a^	0 (0.0)	0 (0.0)	0 (0.0)	0 (0.0)	0 (0.0)	0 (0.0)	1 (25.0)	0 (0.0)	2 (40.0)	3 ( 8.8)
Constipation, n (%)^a^	0 (0.0)	2 (50.0)	0 (0.0)	0 (0.0)	0 (0.0)	0 (0.0)	0 (0.0)	0 (0.0)	0 (0.0)	2 (5.9)
Depression, n (%)^a^	0 (0.0)	0 (0.0)	0 (0.0)	0 (0.0)	0 (0.0)	0 (0.0)	0 (0.0)	0 (0.0)	2 (40.0)	2 (5.9)
**Laboratory abnormalities**										
Anemia^b,c^, n (%)	0 (0.0)	0 (0.0)	0 (0.0)	0 (0.0)	0 (0.0)	0 (0.0)	0 (0.0)	0 (0.0)	1 (20.0)	1 (2.9)
Decreased hemoglobin^d^, n (%)	0 (0.0)	0 (0.0)	0 (0.0)	0 (0.0)	0 (0.0)	0 (0.0)	0 (0.0)	0 (0.0)	1 (20.0)	1 (2.9)
Decreased absolute lymphocyte count^d^, n (%)	0 (0.0)	0 (0.0)	0 (0.0)	0 (0.0)	0 (0.0)	0 (0.0)	0 (0.0)	0 (0.0)	1 (20.0)	1 (2.9)
Elevated aspartate aminotransferase value^e^, n (%)	0 (0.0)	1 (25.0)	0 (0.0)	0 (0.0)	0 (0.0)	0 (0.0)	0 (0.0)	0 (0.0)	0 (0.0)	1 (2.9)
Hyperglycemia^b^, n (%)	0 (0.0)	0 (0.0)	0 (0.0)	0 (0.0)	1 (25.0)	0 (0.0)	0 (0.0)	0 (0.0)	0 (0.0)	1 (2.9)
Hematuria^b^, n (%)	0 (0.0)	0 (0.0)	0 (0.0)	0 (0.0)	0 (0.0)	0 (0.0)	1 (25.0)	0 (0.0)	0 (0.0)	1 (2.9)

^a^Preferred term according to the Medical Dictionary for Regulatory Activities version 14.0.

^b^Laboratory abnormality reported as a TEAE.

^c^Grade 1 in severity.

^d^Grade 3 in severity.

^e^Grade 2 in severity.

*TEAE* treatment-emergent adverse event.

Note: Subjects were counted only once for each preferred term, regardless of how many events the subject reported.

Three subjects (8.8%) reported 4 treatment-emergent SAEs (TESAEs): 1 subject in the 3.0 mg/kg single-dose group (osteomyelitis and skin ulcer), 1 subject in the 5.0 mg/kg/week multiple-dose group (vertigo), and 1 subject in the 1.0 mg/kg/week multiple-dose group (chronic myelogenous leukemia [CML]). Of these, only CML, with onset 10 months after study completion, was considered possibly related to treatment. Two subjects in the 5.0 mg/kg/week multiple-dose group discontinued treatment: 1 treatment discontinuation was due to TEAEs of head injury and vertigo, and the other due to a non-TESAE of normochromic normocytic anemia, with onset prior to administration of the first MEDI-546 dose. Both events were considered unrelated to treatment. There were no deaths in this study.

Six subjects had abnormal laboratory results, with 3 abnormalities reported as TEAEs (Table 2). Of the 6 subjects, 4 also had abnormal values at baseline and 4 had a history of similar findings. Most events were transient and resolved by the following evaluation visit. One laboratory abnormality of low red blood cell count, reported as a grade 1 TEAE of anemia, was considered possibly related to treatment.

Infections occurred in 14 subjects (41.2%). All infections were grade 1 or 2 in severity, except for 1 grade 3 osteomyelitis in the 3.0 mg/kg single-dose group that lasted 8 days and was considered remotely related to treatment. One subject in the 5.0 mg/kg/week multiple-dose group reported a grade 1 infusion-related reaction (pruritus and urticaria) that required temporary (48 minutes) interruption of dosing and was judged as probably related to MEDI-546.

Viral surveillance showed a low percentage of subjects converting from normal test results at baseline to abnormal results post MEDI-546 administration: 4/31 subjects (12.9%) for EBV and 2/24 subjects (8.3%) for HPV. There were no abnormal CMV, HSV-1, or HSV-2 test results throughout the study. All 7 subjects with a history of viral reactivation with HSV tested negative after receiving MEDI-546. One subject in the 20.0 mg/kg single-dose group with a history of viral reactivation developed oral herpes (grade 1) 52 days after dosing. The infection lasted 15 days and the investigator determined it to be unrelated to treatment.

### Pharmacokinetics

**Figure 2:**
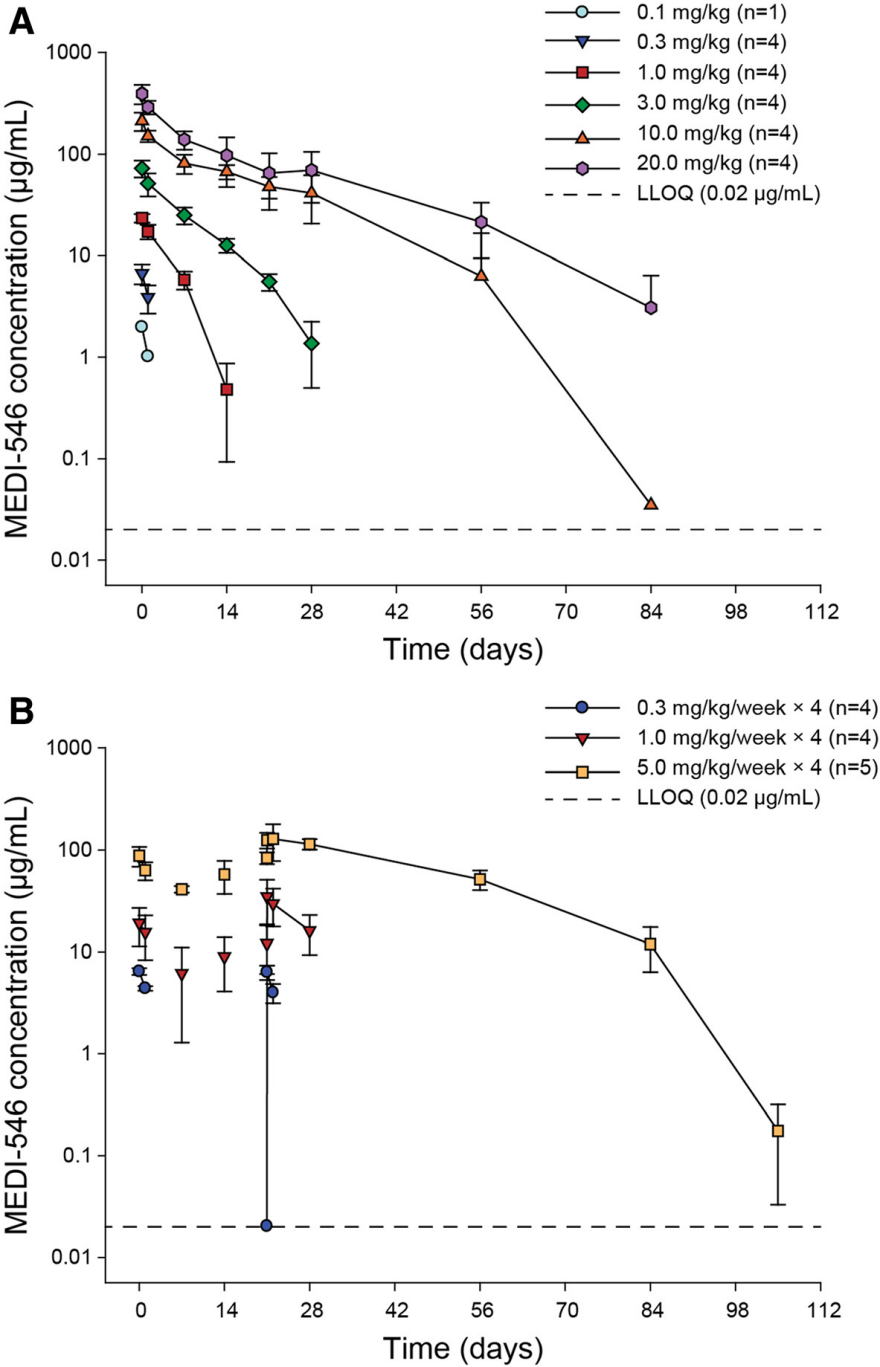
**Mean (±SD) serum MEDI-546 concentration. A**. Single IV Administration; **B**. Multiple IV Administrations. LLOQ, lower limit of quantitation; SD, standard deviation. Mean data below LLOQ are not plotted.

After single dose administration, MEDI-546 exhibited non-linear PK at lower dose levels (<10.0 mg/kg) (Figure 2A, Table 3). Mean AUC_inf_ increased more than proportionally with MEDI-546 dose from 0.1 to 10.0 mg/kg, and proportionally from 10.0 to 20.0 mg/kg. Systemic clearance decreased from 40.8 to 4.67 mL/kg/day when the dose was increased from 0.1 to 20.0 mg/kg. MEDI-546 terminal t_1/2_ was more prolonged at higher doses. At the highest dose level investigated (20.0 mg/kg) the terminal t_1/2_ was 11.8 days.

**Table 3:** Noncompartmental pharmacokinetic parameters

**Parameter**	**MEDI-546**											
	**0.1 mg/kg single dose**	**0.3 mg/kg single dose**	**1.0 mg/kg single dose**	**3.0 mg/kg single dose**	**10.0 mg/kg single dose**	**20.0 mg/kg single dose**	**0.3 mg/kg/week multiple dose**		**1.0 mg/kg/week multiple dose**		**5.0 mg/kg/week multiple dose**	
							**1**^**st **^**dose**	**4**^**th **^**dose**	**1**^**st **^**dose**	**4**^**th **^**dose**	**1**^**st **^**dose**	**4**^**th **^**dose**
Number of subjects	1	4	4	4	4	4	4	4	4	4	5	3
C_max_, mean (SD), μg/mL	1.97 (NA)	6.69 (1.47)	23.3 (2.22)	72.4 (13.7)	213 (44.0)	394 (83.5)	6.43 (0.470)	6.32 (1.01)	19.1 (7.80)	35.8 (15.5)	87.6 (19.3)	135 (26.5)
AUC^a^, mean (SD), μg · day/mL	2.45 (NA)	12.4 (4.91)	102 (14.1)	497 (105)	2610 (728)	4870 (1750)	NA	10.7 (2.53)	NA	159 (68.9)	NA	849 (165)
CL, mean (SD), mL/kg/day	40.8 (NA)	27.4 (11.0)	9.94 (1.44)	6.26 (1.43)	4.07 (1.13)	4.67 (2.18)	NA	NA	NA	NA	NA	NA
t_1/2_, mean (SD), d	0.84 (NA)	1.24 (0.358)	2.96 (0.593)	4.07 (1.23)	7.70 (2.26)	11.8 (2.06)	NA	1.06 (0.174)	NA	4.94 (1.47)	NA	6.28 (0.921)

^a^AUC_inf_ for single-dose groups and AUC_0–7_ for multiple-dose groups.

AUC_inf_ area under the serum concentration-time curve from zero to infinity; AUC_0–7_, AUC from day 0 to day 7 post dose administration; CL, systemic clearance; C_max_, maximum serum concentration; d, days; NA, not available; SD, standard deviation; t_1/2_, serum elimination half-life.

In the multiple-dose groups, non-linear PK was also observed at lower doses (<1.0 mg/kg/week). AUC_0–7_ was more than dose proportional to MEDI-546 dose from 0.3 to 1.0 mg/kg/week, and proportional from 1.0 to 5.0 mg/kg/week (Figure 2B, Table 3). Mean t_1/2_ ranged from 1.1 to 6.3 days across the multiple dose range 0.3 to 5.0 mg/kg/week. After 4 weekly doses, steady-state was not reached, as shown by increasing trough (pre-dose) concentrations.

### Immunogenicity

Two of 21 subjects (9.5%) in the single-dose groups and 3 of 11 subjects (27.3%) in the multiple-dose groups tested positive for ADAs following MEDI-546 administration. Most positive titers were low (≤1:30) and transient, detectable only at 1 or 2 study visits. One subject in the 0.3 mg/kg/week multiple-dose group had persistent positive titers of 1:240–1:960 between study days 28 and 105. Serum MEDI-546 levels for subjects who were positive for ADA did not appear to differ from those of subjects who were negative for ADA, in the same dose groups.

### Pharmacodynamics

**Figure 3:**
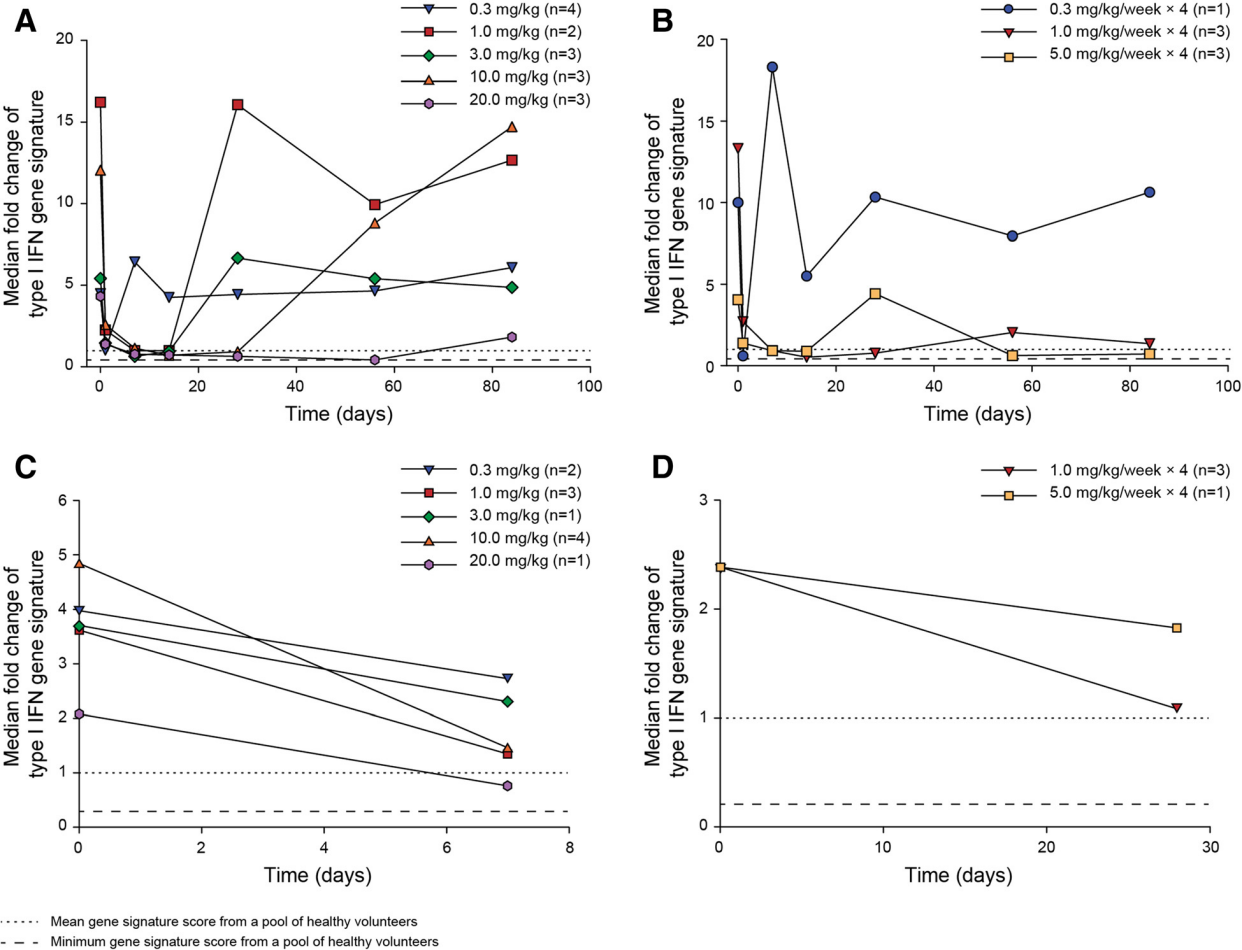
**Median fold change of type I IFN gene signature by visit for subjects with positive baseline gene signature. A**. In Whole Blood in Single-Dose Groups; **B**. In Whole Blood in Multiple-Dose Groups; **C**. In Skin in Single-Dose Groups; **D**. In Skin in Multiple-Dose Groups. IFN, interferon.

At baseline, 22 and 15 subjects had positive type I IFN gene signature score in whole blood and skin, respectively. Subjects with elevated baseline signature in whole blood who received MEDI-546 had suppressed gene signature; the effect seemed to be greater when the amount of drug was increased. All groups reached or approached gene signature peak inhibition at day 1 post-dose (Figures 3A and 3B).

Of the 22 subjects with a positive baseline gene signature in whole blood, 15 were in the single-dose groups and 7 in the multiple-dose groups. In single-dose groups ≥1.0 mg/kg, 11 subjects with positive baseline signature score in whole blood had ≥70% inhibition of the signature at day 14. Persistent decreases until day 84 were observed in 2 of 3 subjects in the 20.0 mg/kg group (Figure 3A). In multiple-dose groups, ≥1.0 mg/kg/week, 5 of 6 subjects had ≥70% inhibition at day 14. The inhibition persisted until day 84 in 2 of 3 subjects in the 1.0 mg/kg/week group and both subjects in the 5 mg/kg/week group (Figure 3B).

Of the 15 patients with a positive baseline skin signature, 11 were in the single-dose groups and 4 in the multiple-dose groups. In single-dose groups ≥1.0 mg/kg, MEDI-546 decreased the expression of type I IFN-inducible genes in skin at day 7 (Figure 3C). In multiple-dose groups ≥1.0 mg/kg/week, expression was decreased at day 28 (Figure 3D). Five of 11 patients in the ≥1.0 mg/kg single-dose cohorts and all 3 patients in the 1.0 mg/kg/week multiple-dose cohort had ≥70% inhibition.

## Discussion

This is the first-time-in-human study of MEDI-546, a human monoclonal antibody targeting the IFN-mediated pathway in SSc. MEDI-546 was administered as single (0.1–20.0 mg/kg) and multiple (0.3–5.0 mg/kg/week) escalating IV doses in adult subjects with SSc, who had at least moderate skin thickening. As MEDI-546 binds to a novel target (IFNAR1) that plays a major role in the IFN pathway, the starting dose of 0.1 mg/kg was recommended based on a translational modeling and simulation approach to predict the minimum anticipated biological effect level in humans. The unoccupied IFNAR was expected to return to near baseline levels 1–2 days following a single administration of 0.1 mg/kg MEDI-546.

In this study, MEDI-546 appeared to have an adequate safety and tolerability profile. Most TEAEs were mild or moderate and were reported at similar frequencies across the dose groups. The most frequent TEAEs were upper respiratory tract infection, headache, diarrhea, and nausea. One infusion-related reaction (grade 1) was reported in the 5.0 mg/kg/week multiple-dose group and resolved after treatment with antihistamine and temporary interruption of dosing. One subject in the 1.0 mg/kg/week multiple-dose group had a TESAE of CML, with onset approximately 10 months after completing the study. The event was considered possibly related to MEDI-546, with alternate etiologies of genetic predisposition, or chemical, drug, or radiation exposure. As this was a short-term study, the significance of this finding is unclear and will be monitored in further studies across SSc and other diseases. Even though there is evidence suggesting that the type I IFN pathway is involved in tumor surveillance [28,29], to date, IFN blockade has not been associated with CML, and there have been no reports of spontaneous tumor formation in IFNAR-deficient mice.

The type I IFN system plays a critical role in limiting the spread of viral infections. As such, inhibition of type I IFN-mediated signaling may result in increased susceptibility to infections, including reactivated latent infections. In this study, 41.2% of subjects developed infections, most of which were grade 1 or 2 in severity, including one grade 3 event of osteomyelitis that was considered remotely related to MEDI-546 treatment. The proportions of subjects who had negative viral surveillance test results at baseline that changed to positive after MEDI-546 treatment were low: 4/31 subjects for EBV and 2/24 subjects for HPV. Positive baseline viral test results for HSV reverted to negative for all subjects.

It is uncertain whether HPV reactivation in the 2 subjects mentioned above was related to immunosuppression from investigational product, infection during the course of the study or as a result of a potential false positive result arising from analytical inaccuracy of the HC2 assay. The College of American Pathologists recently warned that limitations of the HC2 assay can lead to 5% false-positive results when no HPV DNA is present [30].

Positive EBV tests for the 4 subjects occurred only on a single study day and showed no association with dose strength or frequency. In addition, changes in these results were not associated with any trends in worsening of clinical symptoms. Furthermore, positive baseline viral test results for HSV reverted to negative for all subjects. One subject with positive EBV levels at baseline that became negative after initiation of MEDI-546 treatment also showed suppression of the gene signature. Although no increased risk of viral infections with MEDI-546 was identified in this study, viral reactivation events are important to monitor, and they have been defined as adverse events of special interest that are being closely monitored in other studies.

As with other monoclonal antibodies targeting cell-membrane associated receptors, the PK of MEDI-546 was subject to the target-mediated clearance mechanism (antigen sink effect) [31-34]. At lower doses (single doses <10.0 mg/kg and multiple doses <1.0 mg/kg/week), MEDI-546 exhibited non-linear PK. The non-linear elimination pathway presumably involves the binding of MEDI-546 to IFNAR and the subsequent internalization and intracellular degradation of the antibody-receptor complex. This resulted in a more rapid systemic clearance and shorter elimination half-life at lower doses. At higher doses (single doses ≥10.0 mg/kg and multiple doses ≥1.0 mg/kg/week), IFNAR was presumably fully occupied and the PK of MEDI-546 became dose-proportional, with a more prolonged half-life. The systemic clearance of MEDI-546 decreased with dose. The presence of ADAs was detected in 5 subjects (15.6%), but generally, positive titers were low and transient. The presence of ADAs had no apparent impact on MEDI-546 serum PK levels.

MEDI-546 decreased type I IFN gene expression in whole blood and skin for subjects with positive scores at baseline. Expression of type I IFN inducible genes in whole blood reached or approached peak inhibition within 1 day after dosing and persisted through day 84 in the multiple-dose groups. Although only small numbers of subjects received MEDI-546, there seemed to be a dose-dependent neutralization of the type 1 IFN gene signature in whole blood. These results support further investigation of MEDI-546 as a key molecule that inhibits IFN gene expression in the treatment of SSc.

Our study had several limitations that should be taken into account in the interpretation of these results. This was an early phase study, with relatively low numbers of subjects in each treatment group. Effect of MEDI-546 on measures of disease activity and patient-reported outcomes were not determined because of the open-label, non-placebo-controlled study design and small sample size. The gene signature suppression should also be interpreted with both patient-to-patient molecular variability and modest sample sizes (i.e. n = 1 to 4 samples maximum) in mind. In lieu of error bars, the median for each cohort is provided (Figure 3) to illustrate the primary trend in gene signature suppression within each cohort. Results should also be interpreted in light of the inherent limitations of non-randomized, open-label, uncontrolled studies [35]. Larger, longer-term studies are needed to establish any relationship between the inhibition of IFN-mediated signaling and the potential occurrence of malignancies. Caution is advised in extrapolating safety data from this study to other patient populations, as SSc is associated with a high prevalence of comorbidities [36].

## Conclusions

This Phase 1 study of MEDI-546 at single (0.1–20.0 mg/kg) and multiple (0.3–5.0 mg/kg/week, 4 doses) IV doses had an adequate safety and tolerability profile in adults with SSc. Administration of MEDI-546 resulted in sustained inhibition of the type I IFN gene signature. This study supports the continued clinical development of MEDI-546 in subjects with SSc or other IFN-related inflammatory diseases. Further clinical studies are currently ongoing (NCT01559090, NCT01438489).

## Abbreviations

ADA: Anti-drug antibody; AE: Adverse event; CML: Chronic myelogenous leukemia; CMV: Cytomegalovirus; EBV: Epstein-Barr virus; ECL: Electrochemiluminescent; HC2: Hybrid capture 2 High-Risk assay; HPV: Human papilloma virus; HSV: Herpes simplex virus; IFN: Interferon; IFNAR: Interferon-α receptor; IV: Intravenous; mRSS: Modified rodnan skin score; PD: Pharmacodynamics; PK: Pharmacokinetics; SAE: Serious adverse event; SSc: Systemic sclerosis; TEAE: Treatment-emergent adverse event; TESAE: Treatment-emergent serious adverse event.

## Competing interests

This study was funded by MedImmune. Research funding for the conduct of this study was received by all investigators or their institutions. YY, NE, LC, BW, and SY are employees of MedImmune; YY and SY own stock in AstraZeneca.

## Authors’ contributions

AG, SLP, TG, ES, and SY participated in the acquisition, analysis and interpretation of the data and provided critical review of manuscript. TF, VH, and RWS participated in the acquisition of data and provided critical review of manuscript. YY participated in the study design and analysis of the pharmacodynamic data and provided critical review of manuscript. BW participated in the study design, analysis of pharmacokinetics data and provided critical review of manuscript. NE participated in the design and analysis of the study and provided critical review of manuscript. LC was involved in the analysis of the pharmacokinetic data and provided critical review of manuscript. All authors read and approved the final manuscript.
